# Frequency of sarcopenia and associated outcomes in patients with chronic obstructive pulmonary disease

**DOI:** 10.3906/sag-1909-36

**Published:** 2020-08-26

**Authors:** Havva DEMİRCİOĞLU, Fatma Gökşin CİHAN, Ruhuşen KUTLU, Şebnem YOSUNKAYA, Adil ZAMANİ

**Affiliations:** 1 Department of Family Medicine, Meram Faculty of Medicine, Necmettin Erbakan University, Konya Turkey; 2 Department of Chest Diseases, Meram Faculty of Medicine, Necmettin Erbakan University, Konya Turkey

**Keywords:** Chronic obstructive pulmonary disease, dyspnoea, sarcopenia

## Abstract

**Background/aim:**

We aimed to evaluate the prevalence of sarcopenia and associated outcomes in patients with chronic obstructive pulmonary disease (COPD).

**Materials and methods:**

This cross-sectional study was performed on 219 patients aged 50 years and over who were diagnosed with chronic obstructive pulmonary disease (COPD) according to the Global Initiative for Chronic Obstructive Lung Disease (GOLD) guidelines. The study included 196 (89.5%) male and 23 (10.5%) female patients. The mean age of the patients was 66.9 ± 10.1 years. To diagnose sarcopenia, muscle function was determined by a gait speed test. Muscle strength was assessed with a hand dynamometer and muscle mass was measured with a bioelectrical impedance analysis device. Pulmonary function tests and six-min walking tests were also performed. The modified Medical Research Council (mMRC) dyspnoea scale was used to evaluate all the participants. Our sample consisted of sarcopenic patients at different stages (17 presarcopenic patients (7.8%), 32 patients with sarcopenia (14.6%), 65 patients with severe sarcopenia (29.7%), and 105 nonsarcopenic patients (47.9%).

**Results:**

Sarcopenia was significantly associated with age, BODE (body mass index (BMI), airflow obstruction, dyspnoea, and exercise capacity) index, GOLD spirometric classification, mMRC dyspnoea scale score, BMI, and educational status. Sarcopenia in COPD patients was firmly related to the severity of the disease and its prognosis. The prevalence of sarcopenia increased in severe and very severe COPD cases. The dyspnoea score was higher, and exercise capacities were lower in sarcopenic patients.

**Conclusions:**

Sarcopenia in COPD patients was closely related to the severity of COPD and a negative prognosis. The frequency of sarcopenia increased in severe and very severe COPD cases. Dyspnoea scores were higher and exercise capacities were lower in patients with sarcopenia. In patients with COPD, a diagnosis of sarcopenia should be considered, and preventive measures should be taken before irreversible changes develop.

## 1. Introduction

The current definition of sarcopenia describes the condition as one which involves the loss of muscle capacity, muscle mass, and functional capacity [1]. It is assumed that sarcopenia is a consequence of hormonal and immunological changes that occur on account of ageing. Nevertheless, recent studies have shown that chronic inflammatory disorders also cause sarcopenia [2]. Studies have shown that the loss of muscle increases in individuals with chronic inflammatory diseases due to increased levels of proinflammatory cytokines, particularly TNF-α and IL-6.

Chronic obstructive pulmonary disease (COPD) develops as a result of inflammatory processes working against harmful gases and inhaled particles, especially cigarette smoke. The systemic effects of the inflammatory process that occurs in the lungs can explain the close relationship between COPD and comorbidities [3]. Acute inflammation in the lung periphery leads to an increase in acute phase proteins such as C-reactive protein, fibrinogen, serum amyloid A, and surfactant protein D by driving the transmission of cytokines such as TNF-α, IL-1β, and IL-6 into systemic circulation. This increase becomes more evident through exacerbations. Systemic inflammation causes skeletal muscle atrophy and cachexia, initiates comorbid conditions, and increases their severity.

There are significant changes in muscle strength, morphology, mass, oxidative capacity, and resistance in severe and very severe COPD cases. At this point, muscles contract slowly but get tired quickly, resulting in early lactic acidosis and reduced exercise capacity. Prolonged immobilisation, disuse of muscles, hypoxemia, hypercapnia, acidosis, malnutrition, and systemic steroid use are also responsible for functional and structural changes in muscles as well as systemic inflammation. Loss of muscle strength and endurance results in fatigue, reduced quality of life, and reduced exercise capacity. One study has emphasised that muscle weakness is closely related to morbidity and mortality [4]. Consequently, the loss of muscle mass, function, and strength (in another word, ‘sarcopenia’) occurs as a result of the systemic inflammatory effects of COPD. Thus, in this study, we aimed to evaluate the prevalence of sarcopenia and the associated outcomes in COPD patients.

## 2. Materials and methods

This cross-sectional study was performed on 219 patients aged 50 years and over who were diagnosed with COPD according to the Global Initiative for Chronic Obstructive Lung Disease (GOLD) guidelines. The study was conducted at Necmettin Erbakan University, Meram Faculty of Medicine between October 2017 and January 2018. Ethical approval was obtained from the Ethical Committee of Necmettin Erbakan University (approval date: 16.03.2018; approval number: 2018/1277). The study included 219 COPD patients (196 males: 89.5% ; 23 females: 10.5%) with an average age of 66.9 ± 10.1 years.

### 2.1. Anthropometric measurements

The height, body weight, and waist circumference of each participant were measured and recorded. Height was measured to the nearest 0.1 cm using a standard stadiometer (Seca GmbH & Co. KG., Hamburg, Germany) with the participant wearing no shoes. Weight, body fat percentage, muscle mass, basal metabolism, and the amount of visceral fat of all the participants were measured with a bioelectrical impedance analysis (BIA) device. Body mass index (BMI) was calculated with the formula BMI = weight (kg) / height (m2). Skinfold thickness was measured with a digital skinfold analyser device. All the patients were measured in a standing position. The measurements were performed vertically at a distance of 2 cm to the right of the umbilicus.

### 2.2. COPD-related parameters

The patients were asked about the duration of their COPD diagnosis, the number of exacerbations they had in the previous year, the medications they used, and whether they had any comorbidities. Additionally, the participants were asked whether they had ever smoked. Then, the patients were divided into three groups: ‘never smokers’, ‘current smokers’, and ‘former smokers’. The total number of packs smoked per year was calculated for each patient. 

### 2.3. Dyspnoea scales

The dyspnoea levels of the participants were graded from 0 to 4 using the modified British Medical Research Council (mMRC) scale. 

### 2.4. BODE index

The BODE index was calculated with the sum of the scores obtained as a result of the measurement of four factors: BMI (B), airflow obstruction (O), dyspnoea (D), and exercise capacity (E). 

### 2.5. Pulmonary function tests

Pulmonary function tests were performed in the Respiratory Function Laboratory in the Chest Diseases Department of Necmettin Erbakan University Meram Faculty of Medicine. Pulmonary function tests were performed according to the ATS/ERS criteria using standardised equipment (EasyOne Pro® LAB, ndd Medical Technologies, Inc., Andover, MA, USA) [5].

### 2.6. Diagnosis of sarcopenia

After testing muscle mass, gait speed and handgrip strength, the patients were divided into four groups according to the European Working Group on Sarcopenia in Older People (EWGSOP) stages, including ‘presarcopenia’, ‘sarcopenia’, ‘severe sarcopenia’ and ‘nonsarcopenia’. Presarcopenia is defined as low muscle mass with normal muscle strength and physical performance; sarcopenia is defined as low muscle mass with low muscle strength or low physical performance; and severe sarcopenia occurs when all three criteria of sarcopenia (low muscle mass, low muscle strength, and low physical performance) are met.

#### 2.6.1. Evaluation of muscle strength

The handgrip strength measurement of the patients was performed with the Takei grip strength dynamometer. Three consecutive measurements were performed on the dominant hand, and the highest value was recorded for each patient.

#### 2.6.2. Evaluation of muscle mass

**Figure 1:**
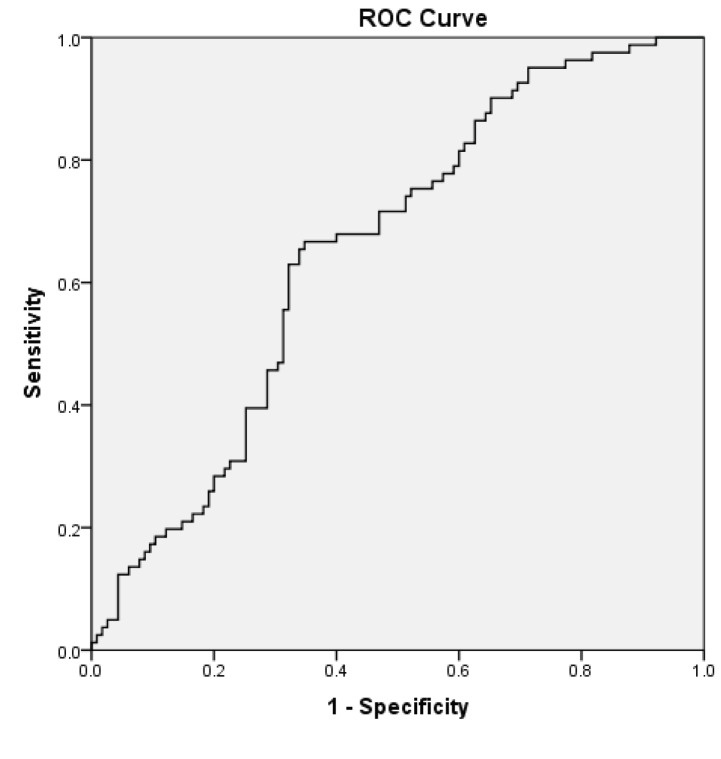
ROC curve and AUC for male patients’ SMMI (AUC = 0.652, P = 0.005; 65.4% sensitivity and 65.2% specificity). AUC = Area under the curve, SMMI = Skeletal muscle mass index, ROC = Receiver operating characteristic curve.

**Figure 2:**
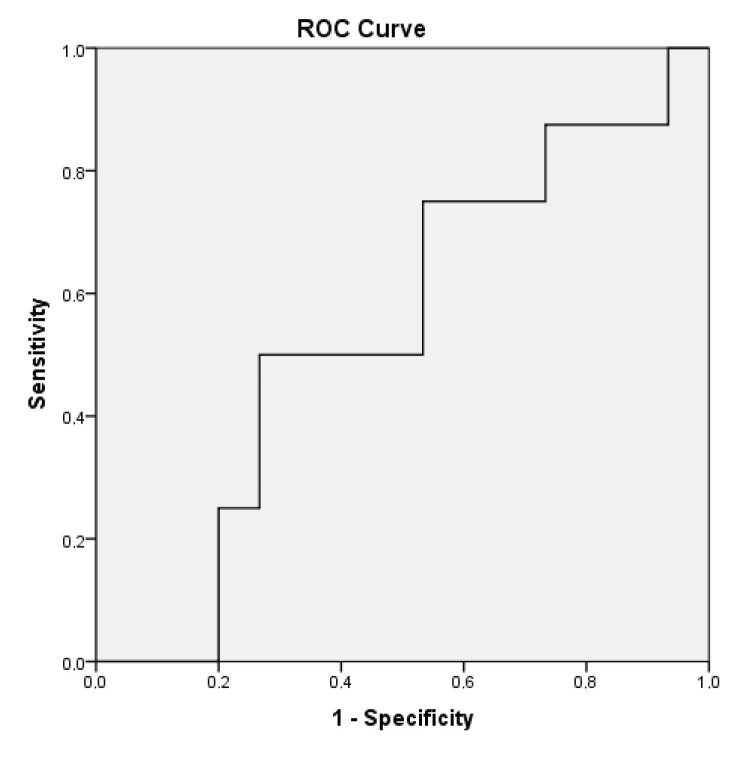
ROC curve and AUC for female patients’ SMMI (AUC = 0.542, P = 0.005; 50.0% sensitivity and 46.7% specificity). AUC = Area under the curve, SMMI = Skeletal muscle mass index, ROC = Receiver operating characteristic curve.

The body composition of each patient was determined with the BIA method using the Tanita InnerScan BC-532 Body Composition Monitor (Tokyo, Japan). The measurements were taken while the patients were fasting with no urge to urinate and wearing no metal items (e.g., necklace, ring, or watch). Muscle mass was automatically calculated by the BIA device. Additionally, the BIA device also measured lean body mass (LBM). Skeletal muscle mass (SMM) (kg) was calculated with the formula of [0.566 ×* LBM] [6]. Then, the skeletal muscle mass index (SMMI) (kg/m^2^ was calculated by dividing the SMM by the square of the patient’s height. A receiver operating characteristics (ROC) analysis was performed to determine the cut-off values of the SMMI. Values less than 9.84 kg/m2 for females and under 10.37 kg/m^2^ for males were considered to be indicative of low muscle mass (Figure 1, 2).

#### 2.6.3. Evaluation of gait speed

The subjects were instructed to walk a distance of 6 metres in their regular walking style in order to determine their gait speed (6-MWT). This walking time was measured using a digital stopwatch. At the start, each participant was located just behind the starting point. After the entire body exceeded the starting point, the time measurement started. The time measurement terminated when the entire body crossed the end point. The speed was calculated as the distance divided by the walking time. The gait speed was measured twice, and the mean of the two trials was used. According to the EWGSOP diagnostic algorithm, the patients with a gait speed lower than 0.8 m/s were considered to have a low gait speed [7]. 

### 2.7. Statistical analysis

Statistical analysis was performed with the Statistical Package for the Social Sciences software (SPSS version 20, IBM Corp., Armonk, NY, USA). The tables show the mean and standard deviation (SD) values as descriptive statistics of the continuous variables, and the frequency (χ2) and percentage (p) values for the descriptive statistics of the categorical data. The independent samples t-test and one-way ANOVA tests were used for the comparison of the quantitative data, and a chi-squared test was performed to compare the categorical data. The effects of factors associated with sarcopenia were evaluated using univariate and multivariate regression analysis. A ROC analysis was performed to determine the male and female cut-off values of skeletal muscle mass index (SMMI) and muscle strength. The results were evaluated with a confidence interval of 95%, and the level of significance, P, was set at 0.05. 

## 3. Results

### 3.1. Sociodemographic characteristics of patients: 

With respect to the ages of the patients, 55 (25.2%) were 50–59 years of age, 76 patients (34.7%) were 60–69, 59 patients (26.9%) were 70–79, and 29 patients (13.2%) were ≥80. Of the participants, 49 (22.4%) were single and 170 (77.6%) were married. The patients were divided into 5 groups according to their educational status: 40 participants (18.3%) were illiterate, 134 participants (61.2%) had completed primary school, 26 participants (11.9%) had completed secondary school, 10 participants (4.6%) were high school graduates, and 9 participants (4%) were university graduates. The sociodemographic characteristics of the participants are shown in Table 1.

**Table 1:** The sociodemographic characteristics of the participants.

Sociodemographic characteristics	No sarcopenia		Sarcopenia		χ^2^	p
	N	%	n	%		
Age groups					15.016	0.002
50–59	32	58.2	23	41.8		
60–69	44	57.9	32	42.1		
70–79	22	37.3	37	62.7		
≥80 years	7	24.1	22	75.9		
Sex						
Female	12	52.2	11	47.8	0.043	0.835
Male	93	47.4	103	52.6		
Marital status						
Single	22	44.9	27	55.1	0.104	0.747
Married	83	48.8	87	51.2		
Education status						
Illiterate and primary school graduated	77	44.3	97	55.7	3.934	0.047
Secondary school and upper graduated	28	62.2	17	37.8		

As to the status of tobacco use, 26 (11.9%) participants reported that they were nonsmokers, while 42 (19.2%) were current smokers and 151 (68.9) were former smokers. The duration of smoking was 57.8 ± 29.9 packs/year. When the patients were evaluated for the presence of any additional diseases, it was determined that 37 (16.9%) had diabetes mellitus, 74 (33.1%) had hypertension, 72 (32.9%) had coronary artery disease, and 29 (13.1%) had benign prostatic hyperplasia.

### 3.2. Prevalence of sarcopenia:

The cut-off values for muscle strength were determined for male and female patients via the ROC analysis. The values under 14.25 kilograms for females and 28.75 kilograms for males were considered to be indicative of low muscle strength. The ROC analyses were performed for grip strength by using the cut-off values that predicted a gait speed of <0.8 m/s; for calf circumference, the cut-off values that predicted low muscle mass were used [8]. Table 2 shows the relationship between sarcopenia and some other parameters. Sarcopenic patients were significantly older, and their BMI was also lower. In sarcopenic patients, muscle mass, fat ratio, and skinfold thickness were significantly lower. Our sample consisted of sarcopenic patients at different stages (17 presarcopenic patients (7.8%), 32 patients with sarcopenia (14.6%), 65 patients with severe sarcopenia (29.7%), and 105 nonsarcopenic patients (47.9%). While comparing all the parameters in our study, the patients in the stages of presarcopenia, sarcopenia, and severe sarcopenia were combined and divided into two main groups as those who were sarcopenic and those who were nonsarcopenic. Table 3 indicates the relationship between sarcopenia and the COPD findings. The percentage of FEV1 was significantly lower in the sarcopenic patients compared to the nonsarcopenic patients, and the number of hospitalisations in one year was significantly higher for sarcopenic patients as well. As the BODE index, GOLD stage, and mMRC dyspnoea scale scores increased, the incidence of sarcopenia increased significantly.

**Table 2:** The relationship between sarcopenia and some other parameters.

	No sarcopenia	Sarcopenia	T	P
	Mean ± SD	Mean ± SD		
Age ( year )	64.6 ± 9.1	69.1 ± 10.4	3.366	0.001
Waist circumference (cm)	110.9 ± 15.8	93.7 ± 13.2	–8.736	< 0.001
Weight (kg)	85.4 ± 14.7	62.9 ± 12.1	–12.363	< 0.001
BMI (kg/m^2^)	30.2 ± 5.3	22.7 ± 4.2	–11.636	< 0.001
Muscle mass ( kg )	58.4 ± 7.6	44.2 ± 5.8	–15.644	< 0.001
Fat rate ( % )	26.8 ± 10.4	23.9 ± 10.3	–1.995	0.047
Visceral fat ( kg )	15.1 ± 11.6	10.7 ± 4.8	–4.529	< 0.001
SFT ( mm )	26.3 ± 8.9	19.2 ± 9.1	–5.791	< 0.001

BMI: Body mass index, SFT: Skin-fold thickness.

**Table 3:** The relationship between sarcopenia and COPD findings.

	No sarcopenia	Sarcopenia	T	P
	Mean ± SD	Mean ± SD		
FEV_1_%	47.6 ± 18.5	41.5 ± 16.9	–2.560	0.011
FEV_1_ - L	1.4 ± 0.6	1.1 ± 0.5	–3.735	< 0.001
FVC%	57.0 ± 19.8	52.9 ± 18.8	–1.555	0.121
FVC - L	2.1 ± 0.8	1.8 ± 0.7	–2.819	0.005
FEV_1_ / FVC	63.2 ± 7.4	59.2 ± 8.9	–3.654	< 0.001
mMRC	1.56 ± 1.1	2.3 ± 1.0	5.035	< 0.001
BODE index	3.5 ± 2.7	5.4 ± 2.7	5.140	< 0.001
Diagnosis time (year)	6.9 ± 7.5	7.4 ± 6.7	0.417	0.677
Hospitalizations / year	1.55 ± 2.0	3.25 ± 5.1	3.160	0.002
6-MWT (meter)	308.5 ± 123.4	237.9 ± 105.7	–4.555	< 0.001

Univariate logistic regression analysis was performed with the possible variables that may be associated with sarcopenia. Patient age, BODE index group, GOLD spirometric classification, mMRC dyspnoea scale score, educational status, and BMI were included in the multivariate model because they were related to sarcopenia to a statistically significant extent (P < 0.05). The multivariate logistic regression results are shown in Table 4, and Table 5 shows the correlations between sarcopenia and some of the relevant parameters. The probability of sarcopenia in patients 80 years of age and over was 2611 times higher than for those in the 50–59 age group [OR = 2611, 95% CI; (0.657–10.379), P = 0.173]. Those with FEV1 < 30 were 3740 times more likely to have sarcopenia than those with FEV1 ≥ 80 [OR = 3740, 95% CI; (0.318-–3.988), P = 0.294]. The likelihood of sarcopenia for those with a BODE index of 7–10 was 34,392 times higher than those with a BODE index of 0–2 [OR = 34,392, 95% CI; (4,422–21,160), P = 0.001]. The probability of sarcopenia for those in the group with mMRC dyspnoea scale scores >2 was 3347 times higher than it was for those in the group with mMRC 0–1 [OR = 3347, 95% CI; (0.754–14.868), P = 0.112]. The nonprimary and primary school graduates were 1409 times more likely to have sarcopenia than it was for those who were secondary school graduates or had higher levels of education.

**Table 4:** Table 4. Multivariate logistic regression results.

Variables	Adjusted OR (Sarcopenia versus no sarcopenia)	95% CI	P
Age groups (year)			
50–59	1		
60–69	0.759	0.308– 1.865	0.548
70–79	2.327	0.811– 6.676	0.116
≥ 80 years	2.611	0.657– 10.379	0.173
GOLD spirometric classification			
Stage 1 FEV_1_ ≥ 80	1		
Stage 2 FEV_1_ 50– 79	3.909	0.426– 35.848	0.228
Stage 3 FEV_1_ 30– 49	2.666	0.267– 26.634	0.404
Stage 4 FEV_1_ < 30	3.740	0.318– 43.988	0.294
BODE classification			
BODE 0– 2	1		
BODE 3– 4	5.520	1.496– 20.362	0.010
BODE 5– 6	11.021	1.560– 77.840	0.016
BODE 7– 10	34.392	4.492– 263.308	0.001
Mmrc			
mMRC 0–1	1		
mMRC ≥ 2	3.347	0.754– 14.868	0.112
BMI (kg/m^2^)			
BMI ≥ 30	1		
BMI < 30	36.340	12.389– 106.594	< 0.001

**Table 5:** Correlation between sarcopenia and some parameters.

		1	2	3	4	5	6	7	8
1.Muscle mass index	r	1							
	P								
2.Gait speed	r	0.232**	1						
	P	0.000							
3.Handgrip strength	r	0.350**	0.553**	1					
	P	0.000	0.000						
4.FEV1 %	r	0.131	0.539**	0.260**	1				
	P	0.052	0.000	0.000					
5.FEV1-L	r	0.223**	0.603**	0.416**	0.857**	1			
	P	0.000	0.000	0.000	0.000				
6.mMRC	r	–0.255**	–0.799**	–0.537**	–0.600*	–0.637*	1		
	P	0.000	0.000	0.000	0.000	0.000			
7.BODE	r	–0.249**	–0.837**	–0.529**	–0.722**	–0.724**	0.899**	1	
	P	0.000	0.000	0.000	0.000	0.000	0.000		
8.6-MWT (meter)	r	0.229**	0.970**	0.550**	0.543**	0.603**	–0.818**	–0.858**	1
	P	0.001	0.000	0.000	0.000	0.001	0.000	0.000	

** Correlation is significant at the 0.01 level (2-tailed).

**Figure 3:**
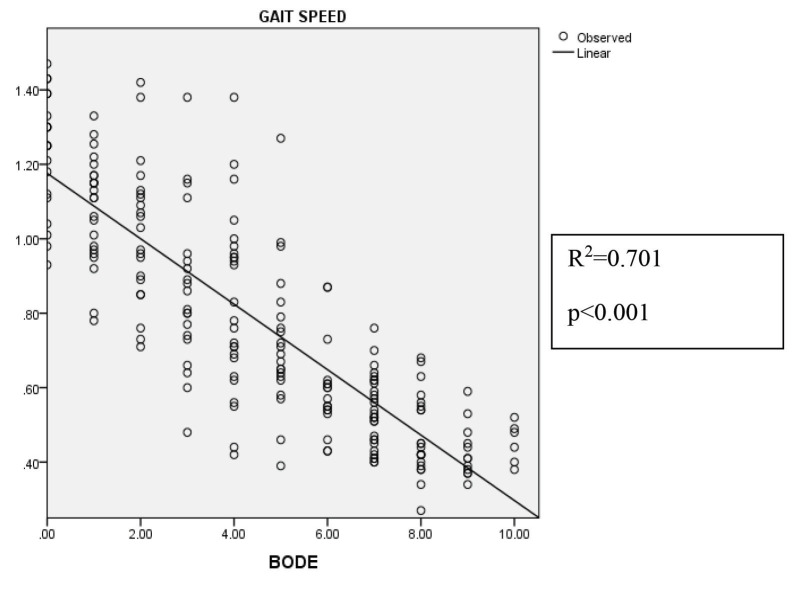
Regression analyses of gait speed and BODE index.

When assessing the linear regression analysis of the gait speed and the BODE index for sarcopenic and non-sarcopenic patients, it was found that 70.1% of the decrease in gait speed could be attributed to the BODE index (R2 = 0.701, P < 0.001) (Figure 3).

## 4. Discussion

This project is a pioneering study in Turkey insofar as it examines sarcopenia in COPD patients using the EWGSOP criteria. Based on the results of this study, body composition and sarcopenia in COPD patients appear to be associated with the severity and prognosis of the disease. The overall prevalence of sarcopenia in 219 COPD patients was found to be 29.7% in our study, and this rate was in accordance with the current literature, in which the reported prevalence is 20%–40%. 

In review of other research on related issues, Costa et al. used a dual-energy X-ray absorptiometry (DXA) for the assessment of the body composition of 96 COPD patients. For the purpose of the densitometric diagnosis of sarcopenia, they used the skeletal muscle index (SMI) calculated as [SMI = ALM/height2 (kg/m2)]. They observed sarcopenia in 36 (39.6%) of the patients [9]. Similarly,, Limpawattana et al. used DXA to assess 121 COPD patients in their study performed in Southeast Asia. The SMI was calculated as ALM/height2. They subjected the patients to a handgrip strength test to measure muscle functions and a 6-MWT. At the end of the study, the prevalence of sarcopenia was determined to be 39.6% [10].

For the diagnosis of sarcopenia in this study, muscle mass was measured with the BIA device, and muscle strength was assessed by using a hand-held dynamometer. A 6-MWT was performed to evaluate physical performance. In our study, the probability of having sarcopenia among those 80 years of age and over was 2.6 times higher than the corresponding rate among those in the 50–59 age group. Costa et al. declared that the prevalence of sarcopenia was 2.8 times higher in those over 67 years, and this rate is closely similar to our results [9]. The prevalence of sarcopenia in some studies has been reported to be 5%–13% for those in the 60–70 age group, 11%–50% for those over 80 years of age, and 8%–40% for those over 60 years of age [11,12]. Akin et al. found that the prevalence of sarcopenia was 63.4% in the geriatric population [13]. In another study conducted on elderly male patients staying in nursing homes, Bahat et al. found that the prevalence of sarcopenia was 85.4% [14].

In our sample of COPD patients, we found that an increased likelihood of being diagnosed with sarcopenia was correlated with an increased BODE index. In fact, the prevalence of sarcopenia in patients who had a BODE index of 0–7 was 34.4 times higher than the rate of patients who had a BODE index of 0–2. Currently, studies on the relationship between sarcopenia and the BODE index in COPD patients are scarce. Nevertheless, two different studies established that there was a strong relationship between the BODE index and the prevalence of sarcopenia [9,15]. Another study suggested that the BODE index was significantly associated with sarcopenia, and that sarcopenia was more prevalent among patients with a worse prognosis [16]. In any event, the BODE index is an important tool for evaluating the prognosis of sarcopenia and predicting mortality in COPD patients. The BODE index can also be used to determine the risk of hospitalisation for patients with COPD.

When the sarcopenia and GOLD spirometric classification were considered together, a significant association was demonstrated between the presence of sarcopenia and disease severity according to the GOLD staging. Jones et al. also reported on this association in their own study, indicating that the prevalence of sarcopenia increased with spirometrically-defined disease severity [17]. Furthermore, in our study, the prevalence of sarcopenia in patients who had FEV1 values of <30 was 3.75 times higher than those who had FEV1 values of ≥80. A significant decrease was also observed in the FEV1 value in sarcopenics and severe sarcopenics in another study [18]. Similarly, Ischaki et al. showed that an increased loss of lean muscle leads to an increase in the severity of disease in COPD patients and, therefore, an increase in the GOLD stage [19]. 

Dyspnoea is one of the most important factors reducing the quality of life and limiting daily life activities in COPD patients [20]. In our study, we examined the relationship between sarcopenia and mMRC dyspnoea scores. Our results showed that the prevalence of sarcopenia increased as the dyspnoea scores increased. The results obtained from other studies on this subject were in line with our results [17,21]. In another study, it was reported that a high mMRC score was also an indicator of poor lung capacity, and this was associated with sarcopenia [10]. Another study identified a correlation between mMRC dyspnoea scale scores and handgrip strength [21]. Since the sarcopenic patients of our study also had a low handgrip strength, similar results were obtained. 

It should be noted that the education level of our patients was low. While illiterate and primary school graduates comprised 79.5% of the sample, only 4% of the patients were university graduates. The findings with respect to this composition were consistent with other studies associating the levels of education and COPD [22]. Additionally, we examined the relationship between educational level and sarcopenia. As far as it is known, there is no study in the literature on the association between educational level and the prevalence of sarcopenia in COPD patients. In our study, it was determined that as the level of education decreased, the prevalence of sarcopenia increased.

The relationship between sarcopenia and BMI was also examined in our study. The prevalence of sarcopenia was higher in the nonobese group. In other words, sarcopenic patients were weaker and generally underweight. Additionally, some level of weakness, lower handgrip strength, and lower waist circumference were expected of sarcopenic patients, and these results were supported by previous studies. A low BMI (<21 kg/m2) is indicative of a poor prognosis on the BODE index, which is a common scoring system used to determine the prognosis of COPD [23]. Accordingly, sarcopenia is closely related to the BODE index, which also predicts a higher risk of mortality. However, the loss of muscle may increase in time, and lost muscle may be replaced by fat stores. Thus, a COPD patient may well have lost considerable lean body mass (LBM) but may have extra fat stores, resulting in an overall normal BMI [24,25]. In another study, it was reported that the loss of skeletal muscle mass is crucially important in the prognosis of COPD patients [4]. A Brazilian study showed that the loss of LBM is associated with a lower BMI in patients with COPD. However, the authors did not find BMI to be associated with COPD severity or a worse prognosis [9]. Nevertheless, some studies have shown that the loss of muscle can reduce survival by up to 50% [25]. This indicates the importance of pulmonary rehabilitation and physical activity in COPD patients.

We have mentioned that the BMI of sarcopenics is low. However, it should not be forgotten that there is a description of what is referred to as ‘sarcopenic obesity’ in the literature. This means that a patient who has a high BMI and a high waist and hip circumference may also suffer from sarcopenia. In some studies, patients with sarcopenia were found to be more overweight and had higher BMIs and waist circumferences than those without sarcopenia [26]. 

In our study, muscle mass index, handgrip strength, and gait speed were significantly correlated with the results of the mMRC dyspnoea scale, BODE index, 6-MWT and pulmonary function tests. In a study performed by Byun et al., low handgrip strength and low SMMI were significantly correlated with mMRC dyspnoea scale scores, COPD Assessment Test (CAT) scores, and 6-MWT times [21] . In a multicentre study, handgrip strength was also found to be significantly correlated with the prognosis of patients with COPD [27]. After the age of 70, a decrease in gait speed and handgrip strength was found to be faster than the loss of muscle mass [28,29]. As a result of these findings, it is clear that the measurements of handgrip strength can provide critical prognostic information about COPD patients.

In a study performed with COPD patients in South Korea, it was reported that sarcopenic patients had shorter 6-MWT times [21] and in other studies, sarcopenia and shorter 6-MWT times were also associated [10,17]. In accordance with these findings, we found that the 6-MWT results of the sarcopenic patients were lower than those of nonsarcopenic patients. Ultimately, the loss of muscle mass in COPD patients leads to a decrease in exercise capacity. This also leads to a decrease in the quality of life indices and an increase in exacerbation frequency and mortality [30]. 

Studies have shown that smoking causes loss of muscle mass by increasing protein catabolism [31]. In our study, no relationship was found between smoking and sarcopenia. Studies conducted on patients with COPD showed that smoking was only associated with a loss of muscle mass, yet it was not related to handgrip strength and gait speed, which are the diagnostic parameters of sarcopenia [32]. There are continued discussions in the literature on this subject, with one study claiming that smoking might only be a risk factor for sarcopenia with other cytokines [7]. However, it should be kept in mind that smoking is the most important risk factor for COPD, and it is the most important trigger of the systemic inflammatory effect of COPD.

In our study, skinfold thickness measurements were lower in the sarcopenic group than in the nonsarcopenic group. In the current literature, there is no relevant study on sarcopenia and skinfold thickness among COPD patients. However, one study performed by Akin et al. in Turkey to determine the cut-off value of sarcopenia in elderly Turkish people found that skinfold thickness was significantly lower in the sarcopenia group [13]. 

Since we performed our study in a tertiary hospital, our study population had more comorbidities and increased disease severity.

## 5. Limitations

There are different methods that can be used to diagnose sarcopenia. In particular, the methods used for measuring muscle mass are very diverse. In our study, we measured muscle mass with a BIA device, as it is portable, takes less time, and is both non-invasive and cheap. Studies have shown that although BIA shows a good reliability and strong correlation with DEXA, it can cause an overestimation of SMI. BIA is also sensitive to the hydration status, temperature, and body position of the participant. Furthermore, although sarcopenia is well-defined, it is still underemphasised in routine clinical management of COPD patients. This study assessed the prevalence of sarcopenia in a group of COPD patients in one health centre. Further multi-centred studies are needed to evaluate sarcopenia in larger COPD patient groups.

## 6. Conclusion

In this study, dyspnoea scores and the prevalence rates of sarcopenia were found to be higher in patients with COPD. The existence of dyspnoea in COPD patients is due to the loss of lung function and also to the loss of peripheral muscle tissue. Depending on the losses of muscle mass, a decrease in exercise capacity, diminished quality of life, and increased mortality can be observed. Thus, a vicious cycle in COPD patients begins after these losses of muscle mass. To improve the quality of life, increase survival, and reduce morbidity in patients with COPD, the necessary precautions against loss of muscle should be taken immediately.

## Acknowledgments

This research did not receive any specific grant or funding from funding agencies in the public, commercial, or not-for-profit sectors. 

## Informed consent

The study was conducted at Necmettin Erbakan University, Meram Faculty of Medicine between October 2017 and January 2018. Ethical approval was obtained from the Ethical Committee of Necmettin Erbakan University (approval date: 16.03.2018; approval number: 2018/1277). Informed consent forms were taken from all the patients.
